# Mortality in Patients With Nonfunctional Adrenal Tumors

**DOI:** 10.1001/jamainternmed.2023.2442

**Published:** 2023-06-26

**Authors:** Jekaterina Patrova, Buster Mannheimer, Jonatan D. Lindh, Henrik Falhammar

**Affiliations:** 1 Department of Clinical Science and Education, Södersjukhuset, Karolinska Institutet, Stockholm, Sweden; 2 Department of Endocrinology, Södersjukhuset, Stockholm, Sweden; 3 Department of Laboratory Medicine, Division of Clinical Pharmacology, Karolinska Institutet, Stockholm, Sweden; 4 Department of Molecular Medicine and Surgery, Karolinska Institutet, Stockholm, Sweden; 5 Department of Endocrinology, Karolinska University Hospital, Stockholm, Sweden

## Abstract

**Importance:**

It is unclear if nonfunctional adrenal adenomas (NFAAs) are associated with increased mortality.

**Objective:**

To analyze mortality and causes of death in patients with NFAA.

**Design, Setting, and Participants:**

A national retrospective register-based case-control study was conducted and included 17 726 patients with a diagnosis of adrenal adenoma in Sweden from 2005 to 2019 who were identified and followed up until death or 2020 as well as 124 366 controls without adrenal adenoma. Individuals with diagnoses indicating adrenal hormonal excess or cancer were excluded. Follow-up started after 3 months of cancer-free survival following the date of the NFAA diagnosis. Sensitivity analyses were performed in subgroups of individuals for whom it was assumed that controls would also have undergone computed tomography: those with acute appendicitis (for whom it was assumed that there was no concern of cancer) and in patients with a combination of gallbladder, biliary tract, and pancreas disorders and 6-month and 12-month cancer-free survival following the date of the NFAA diagnosis. The data were analyzed in 2022.

**Exposures:**

Diagnosis of NFAA.

**Main Outcomes and Measures:**

The primary outcome was all-cause mortality among patients with NFAA after adjustment for comorbidities and socioeconomic factors. Secondary outcomes were mortality due to cardiovascular diseases and cancer.

**Results:**

Among 17 726 cases, 10 777 (60.8%) were women, and the median (IQR) age was 65 (57-73) years; among 124 366 controls, 69 514 (55.9%) were women, and the median (IQR) age was 66 (58-73) years. Among cases, overall mortality during the follow-up period (median, 6.2 years [IQR, 3.3-9.6 years]) was higher compared with controls (hazard ratio [HR] 1.43; 95 CI, 1.38-1.48; adjusted HR [aHR], 1.21; 95% CI, 1.16-1.26). The relative association of NFAA with overall mortality was similar in women and men (aHR, 1.22 [95% CI, 1.15-1.28] vs 1.19 [95% CI, 1.11-1.26]; *P* < .001 in both groups). In contrast, NFAA was associated with a larger increase in mortality among individuals younger than 65 years (aHR, 1.44; 95% CI, 1.31-1.58) than in older individuals (aHR, 1.15; 95% CI, 1.10-1.20; *P* < .001 for interaction). Mortality due to cardiovascular diseases was increased (aHR, 1.21; 95% CI, 1.13-1.29), as was mortality due to cancer (aHR, 1.54; 95% CI, 1.42-1.67). The association between NFAA and mortality remained significant and of similar magnitude in all sensitivity analyses.

**Conclusions and Relevance:**

The results of this case-control study suggest that NFAA was associated with an increased overall mortality and mortality of cardiovascular disease and cancer. The increase was more pronounced among younger individuals.

## Key Points

**Question:**

Is mortality among patients with nonfunctioning adrenal tumors (NFAA) higher than in the general population?

**Findings:**

In this national retrospective register-based case-control study that included 17 726 cases and 124 366 controls, overall mortality among patients with NFAA was higher compared with controls (hazard ratio, 1.43; 95 CI, 1.38-1.48; adjusted hazard ratio, 1.21; 95% CI, 1.16-1.26) during the median follow-up period of 6.2 years (IQR, 3.3-9.6 years).

**Meaning:**

The results of this case-control study suggest that overall mortality among patients with NFAA was higher compared with controls.

## Teaser

This case-control study examines mortality and causes of death in Swedish patients with nonfunctional adrenal adenomas.

## Introduction

The use of abdominal imaging, such as computed tomography and magnetic resonance imaging scans, has resulted in an increased detection of adrenal tumors of unknown origin.^1,2,3^ Different imaging and autopsy series with large patient numbers have reported prevalence of adrenal tumors ranging from 1.05% to 8.7%.^4^ The increasing prevalence over the years is probably associated with the improvement of radiologic techniques and extensive imaging in aging populations.^3^ Adrenal tumors detected on imaging performed for reasons other than suspected adrenal disease or cancer staging are called adrenal incidentalomas (AIs).^3^

Most AIs are benign nonfunctional adrenal adenomas (NFAAs) with no overt hormonal secretion.^3^ However, 7% to 30% of AIs are overproducing small amounts of cortisol without discernable clinical symptoms.^5,6^ This condition is called autonomous cortisol secretion (ACS) but was previously called subclinical Cushing syndrome or subclinical hypercortisolism.^3,7,8^ As diagnostic tests have false-positive rates, the true prevalence of ACS is debated.^9^ Several studies have shown increased risk of obesity,^10^ hypertension, and type 2 diabetes,^11,12,13^ as well as higher mortality risk,^5,9,11,14,15^ associated with cardiovascular diseases^9^ and cancer^15^ among patients with slightly increased cortisol production. It was reported that individuals with normal cortisol levels and AIs exhibit an increased incidence of type 2 diabetes,^1^ dyslipidemia, and hypertension.^16^ However, larger studies supporting this are needed. The aim of this study was to analyze mortality and causes of death in all patients with NFAA in Sweden.

## Methods

### Study Design and Setting

This was a population-based retrospective cohort study. The study protocol was approved by Swedish Ethical Review Authority, and informed consent was not required due to the retrospective nature of the study. By using the unique Swedish personal identity number, data were matched and linked between several national registers. The National Patient Register (all inpatient and specialist outpatient care) data from 1997 to 2019 was used to identify diagnoses, and the Cause of Death Register using data from 2005 until 2020 was used to identify patients who had died as well as causes of death. To analyze potential additional confounders (eTable in Supplement 1), we also used the longitudinal integrated database for health insurance and labor market studies, which comprises detailed individual-level data on socioeconomic factors. All individuals with a first-ever *International Classification of Diseases, Tenth Revision* (*ICD-10*) code of D44.1 (neoplasm of uncertain behavior of the adrenal gland) and/or D35.0 (benign neoplasm of the adrenal gland) from January 1, 2005, to December 31, 2019, were identified. Controls were randomly selected from the total population register and matched by age, sex, and municipality. Individuals with known cancer of any kind (any *ICD-10* C code) diagnosed since 1997 up to 3 months after the first D44.1 or D35.0 diagnosis were excluded. Patients who had received a diagnosis of hormonal activity, such as Cushing syndrome (*ICD-10* code E24), congenital adrenal hyperplasia (*ICD-10* code E25),^17^ primary aldosteronism (*ICD-10* code E26), and pheochromocytoma (*ICD-10* code E27.5), between 1997 and 2019 were excluded from both groups. By excluding all hormonally active lesions, we intended to focus on only NFAA in the study.

Although the matching was performed at the date of NFAA diagnosis, the index date (start of follow-up in the survival analysis) was set at 90 days after the first D44.1 or D35.0 diagnosis to avoid confounding by indication (ie, patients undergoing imaging due to suspected cancer that was then confirmed shortly after the imaging results that revealed the NFAA). To confirm the results, 2 more sensitivity analyses were conducted in which the index date was set to 6 and 12 months after the NFAA diagnosis. The index date in the control group was shifted similarly as cases in the different analyses. As individuals who died or received a cancer diagnosis during these periods (3, 6, and 12 months, respectively, in the different analyses) were excluded, and cases and controls were no longer perfectly matched with regard to age and sex. Thus, these variables were also adjusted for in the multivariable analysis.

We were not able to assemble a control group that was limited to persons who had undergone imaging (eg, computed tomography or a magnetic resonance imagining scan), raising the possibility that the group with the NFAA may have had an imaging examination in pursuit of a cancer diagnosis. To attempt to overcome this limitation, we conducted sensitivity analyses in 2 groups: acute appendicitis (*ICD-10* code K35) or a combination of gallbladder, biliary tract, and pancreas diseases (*ICD-10* codes K80-K87). We looked at cases and controls with acute appendicitis because of the low likelihood that their imaging was due to any concerns about cancer and because we assumed that controls with acute appendicitis also had undergone imaging. We looked at cases and controls with a diagnosis of gallbladder, biliary tract, and pancreas diseases because we would assume that cases and controls underwent an imaging examination to make the diagnosis. Cases were included if they had a K35 or a K80 to K87 diagnosis within 6 months before the D41.1 and/or D35.0 diagnosis. In the sensitivity analyses, the index date for controls was defined by the date of the first-ever K35 or K80-87 diagnosis.

### Statistical Analysis

Descriptive statistics included percentages, means, medians, and IQRs as appropriate. Overall and cause-specific survival probabilities were presented by means of Kaplan-Meier curves. Cases and controls were compared regarding all-cause mortality (primary outcome) as well as death due to cardiovascular diseases or cancers (secondary outcomes) using a Cox proportional hazards ratio analysis with and without adjustment for age, sex, and other potential confounders (eTable in Supplement 1). Given that matching between cases and controls had been done initially in the cohort, we also incorporated matching in the analysis, with each cluster comprising a case and its matched controls, except in 2 of the sensitivity analyses in which this was not possible because controls received new index dates based on K35 or K80 to K87 diagnoses. Age and sex were included as covariates in the regression model to account for any differences between cases and controls. Due to the few individuals in the sensitivity analyses based on K35 or K80 to K87 diagnoses, all longitudinal integrated database for health insurance and labor market studies variables (for which missing values would have been associated with further loss of statistical power) were removed from the model in these analyses. In addition, the variable of previous hospitalization of longer than 3 days was removed because it was inflated by the K35 or K80 to K87 episode before NFAA in cases but not in controls for whom these episodes did not precede the index date. To verify the statistical significance of the differences in adjusted hazard ratios (aHRs) seen in the subgroup analyses, the multivariable analysis of all-cause mortality was repeated post hoc after the addition of interaction terms for being 65 years or older × NFAA and sex × NFAA. The grouping variable of the third subgroup analysis, adrenalectomy, could not be analyzed as an interaction term, since the procedure was not performed in controls without NFAA. The absolute increase in mortality was calculated as the difference in 1-year mortality (total number of deaths divided by total number of years at risk) between cases and controls. Moreover, analysis of the main outcome was repeated in subgroups based on sex, age, and whether adrenalectomy had been performed. *P* < .05 was considered statistically significant. The statistical analysis was conducted in R, version 4.0.3 (R Foundation).

## Results

### Study Population

A total of 17 726 patients with NFAA and with 124 366 controls were included. Of those with NFAA, 10 777 (60.8%) were women and 6949 (39.2%) were men. The median age at the time of the NFAA diagnosis was 65 years (IQR, 57-73 years). In total, 352 of 17 726 patients (2.0%) underwent adrenalectomy. The most common comorbidities in both groups were ischemic heart disease and chronic obstructive pulmonary disease. Table 1 describes medical conditions and socioeconomic factors in the study population at the index date.

**Table 1.** Medical Characteristics and Socioeconomic Factors Among Cases With Nonfunctioning Adrenal Adenoma and Controls at Index Date

Characteristic	No. of cases (%) (n = 17 726)	No. of controls (%) (n = 124 366)
Sex		
Female	10 777 (60.8)	69 514 (55.9)
Male	6949 (39.2)	54 852 (44.1)
Median age (IQR), y	65 (57-73)	66 (58-73)
Diagnosis		
Ischemic heart disease	2529 (14.3)	11490 (9.4)
Chronic obstructive pulmonary disease	1726 (9.7)	3417 (2.7)
Chronic heart failure	1384 (7.8)	4488 (3.6)
Cerebrovascular diseases	1190 (6.7)	5851 (4.7)
Thrombosis	847 (4.8)	2388 (1.9)
Kidney diseases	644 (3.6)	1845 (1.5)
Pancreas diseases	445 (2.5)	938 (0.75)
Liver diseases	432 (2.4)	1000 (0.8)
Inflammatory bowel disease	406 (2.3)	1389 (1.1)
Alcohol misuse	909 (5.1)	3231 (2.6)
Sepsis	57 (0.3)	49 (0.04)
Pneumonia	105 (0.6)	145 (0.1)
Meningitis	4 (0.02)	9 (0.007)
Previous hospitalization >3 d	10 649 (60)	44404 (35.7)
Socioeconomic factors		
Education		
Primary and secondary (<9 y)	3411 (19.2)	22 780 (18.3)
Primary and secondary (9-10 y)	2064 (11.6)	11 528 (9.3)
Upper secondary (<2 y)	5918 (33.4)	36 133 (29)
Upper secondary (3 y)	2007 (11.3)	15 255 (12.3)
Postsecondary (<3 y)	1638 (9.2)	14 141 (11.4)
Postsecondary (≥3 y)	1956 (11)	18 344 (14.6)
Postgraduate	98 (0.6)	1242 (1.0)
Missing	634 (3.6)	4943 (4.0)
Annual income (95% CI), Swedish krona	162 600 (125.300-242 100)	179 800 (130 500-269 100)
Unemployment days	0	0

### All-Cause Mortality

All 142 092 cases and controls entered survival analysis; median follow-up was 6.2 years (IQR, 3.3-9.6 years). Death was confirmed in 3719 of 17 726 cases (21.0%) and in 19 343 of 124 366 controls (15.6%). The overall mortality rate during the follow-up period was increased in patients with NFAA (hazard ratio [HR], 1.43; 95% CI, 1.38-1.48; aHR, 1.21; 95% CI, 1.16-1.26) (Figure, A). The absolute increase in mortality associated with NFAA was 0.95% per year.

**Figure.**
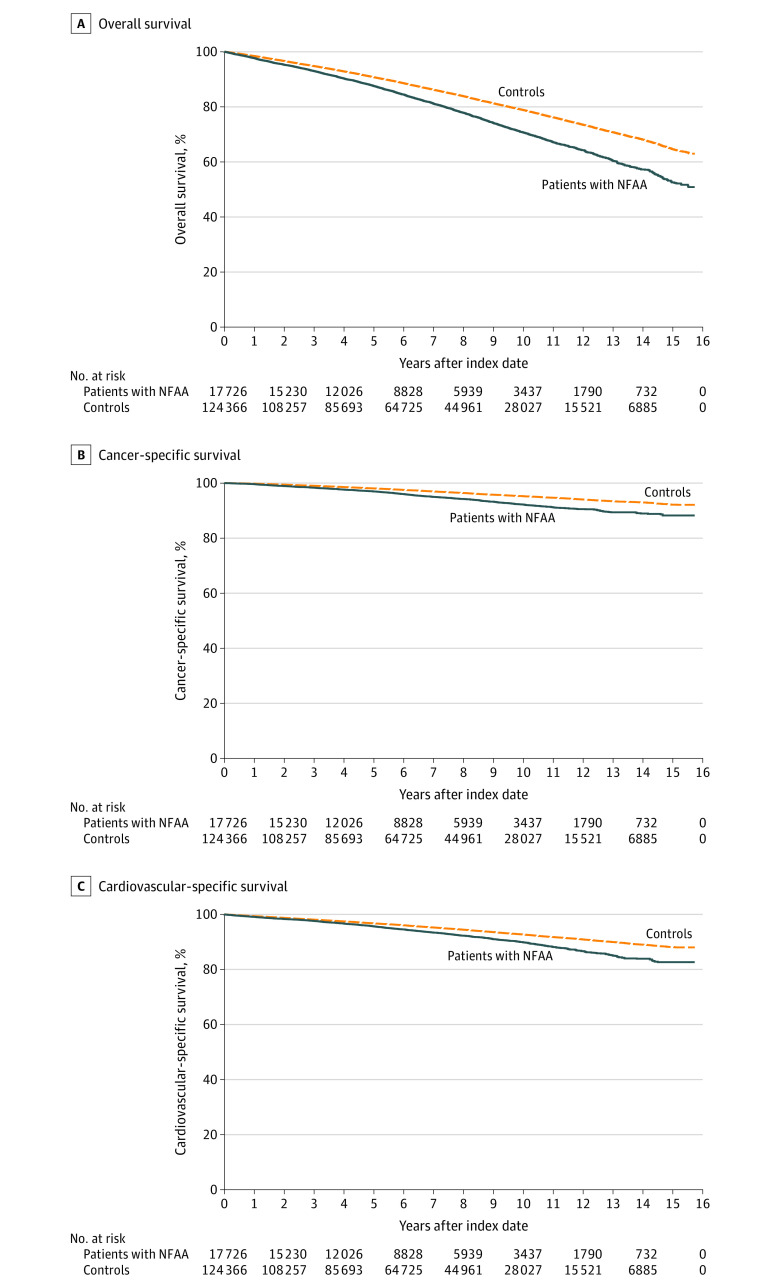
Kaplan-Meier Survival Curves of the Study Participants Kaplan-Meier survival curves showing mortality in 17 726 individuals with nonfunctional adrenal adenomas compared with 124 366 controls. All individuals with cancer discovered during the first 3 months after the diagnosis of the nonfunctional adrenal adenomas have been excluded.

### Cause-Specific Mortality

Table 2 presents the most common causes of death. In total, 851 of all 3719 deaths (22.9%) among patients with NFAA and 3863 of 19 343 deaths (20.0%) among controls were due to cancer. The most common types of cancer deaths in patients with NFAA were lung cancer (248 [29.1%]), pancreas cancer (78 [9.2%]), colon cancer (58 [6.8%]), prostate cancer (37 [4.3%]) and breast cancer (21 [2.5%]). Table 3 and panel B of the Figure show the crude cancer-specific (all types of cancer) survival of individuals with NFAA compared with controls. After adjusting for age, sex, and other potential confounders, cancer-related mortality was increased in patients with NFAA (aHR, 1.54; 95% CI, 1.42-1.67; *P* < .001).

**Table 2.** Causes of Death in Both Groups^a^

Cause of death	Cases, No. (%)	Controls, No. (%)
Cardiovascular diseases	1212 (32.6)	6369 (32.9)
Cancer (all)	851 (22.9)	3863 (20.0)
COPD	295 (7.9)	756 (3.9)
Stroke	165 (4.4)	1121 (5.8)
Dementia	167 (4.5)	1944 (10)
CHF	138 (3.7)	594 (3.1)
Infectious diseases	127 (3.4)	779 (4)
COVID-19	74 (2.0)	366 (1.7)
Sepsis	53 (1.4)	175 (0.9)
Pneumonia	42 (1.1)	339 (1.8)
Trauma	126 (3.4)	746 (3.9)
Liver diseases	59 (1.6)	245 (1.3)
Suicide	36 (1.0)	176 (0.9)
Kidney diseases	25 (0.7)	148 (0.8)
Thrombosis	16 (0.4)	113 (0.6)
Parkinson disease	15 (0.4)	209 (1.1)
Other	60 (1.6)	412 (2.1)
Total	3719	19 343

Abbreviations: CHF, congestive heart failure; COPD, chronic obstructive pulmonary disease.

^a^ All cases with cancer discovered within the first 3 months after the diagnosis of the nonfunctional adrenal adenoma were excluded.

**Table 3.** Mortality Analysis in 17 726 Individuals With Nonfunctional Adrenal Adenomas Compared With 124 366 Controls, Unadjusted and Adjusted to Age, Sex, and Possible Confounders^a^

Characteristic	Cases	Controls	Hazard ratio (95% CI)	*P* value	Adjusted hazard ratio (95% CI)	*P* value
Overall total mortality^b^	3719	19 343	1.43 (1.38-1.48)	<.001	1.21 (1.16-1.26)	<.001
Mortality due to cancer	851	3719	1.63 (1.51-1.76)	<.001	1.54 (1.42-1.67)	<.001
Mortality due to CV diseases	1212	6369	1.43 (1.34-1.52)	<.001	1.21 (1.13-1.29)	<.001
Men	1522	9365	1.35 (1.28-1.43)	<.001	1.19 (1.11-1.26)	<.001
Women	2197	9978	1.52 (1.45-1.59)	<.001	1.22 (1.15-1.28)	<.001
Age <65 y	750	2350	2.26 (2.07-2.46)	<.001	1.44 (1.31-1.58)	<.001
Age >65 y	2969	16 993	1.4 (1.34-1.45)	<.001	1.15 (1.1-1.2)	<.001
Adrenalectomy	352	124 366	0.74 (0.56-0.97)	.03	0.95 (0.72-1.24)	.69
No adrenalectomy	17 374	124 366	1.45 (1.4-1.5)	<.001	1.21 (1.16-1.26)	<.001

Abbreviation: CV, cardiovascular.

^a^ All cases with cancer discovered within the first 3 months after the diagnosis of the nonfunctional adrenal adenoma have been excluded.

^b^ Overall follow-up period up to 16 years.

In total, 1212 of 3719 deaths (32.6%) in patients with NFAA were due to cardiovascular diseases. Table 3 and panel C of the Figure present a Kaplan-Meier survival curve showing mortality due to cardiovascular diseases in individuals with NFAA compared with controls. After adjustment for age, sex, and other potential confounders, mortality due to cardiovascular diseases was increased in individuals with NFAA (aHR, 1.21; 95% CI, 1.13-1.29; *P* < .001).

### Subgroup Analyses

The relative association of NFAA with overall mortality was essentially similar in women and men (aHR: 1.22 [95% CI, 1.15-1.28] vs 1.19 [95% CI, 1.11-1.26]; *P* < .001 in both groups; *P* = 0.20 for interaction) (eFigure 1 in Supplement 1; Table 3). Individuals younger than 65 years with NFAA had more pronounced mortality compared with same-aged individuals in the control group (aHR, 1.44; 95% CI, 1.31-1.58) than those older than 65 years (aHR, 1.15; 95% CI, 1.10-1.20; *P* < .001 in both groups; *P* < .001 for interaction) (eFigure 2 in Supplement 1; Table 3).

Among those who underwent adrenalectomy, mortality was similar to controls (aHR, 0.95; 95% CI, 0.72-1.24; *P* = .69). In contrast, patients with NFAA who did not undergo adrenalectomy had higher overall mortality (aHR, 1.21; 95% CI, 1.16-1.26; *P* < .001) (eFigure 3 in Supplement 1; Table 3).

### Sensitivity Analyses

Among individuals with acute appendicitis, overall mortality remained increased in those with an NFAA (HR, 3.08; 95% CI, 1.93-4.93; aHR, 2.34; 95% CI, 1.44-3.8; *P* < .001 in both). Similar findings were found in patients and controls with gallbladder, biliary tract, and pancreas diseases, whereas those with NFAA had increased mortality (HR, 2.25; 95% CI, 1.92-2.64; aHR 1.32; 95% CI, 1.1-1.59; *P* < .001 in both) (eFigure 4 in Supplement 1).

In the 2 additional sensitivity analyses, the index date was shifted to 6 and 12 months after the NFAA diagnosis and similarly in controls. All-cause mortality was still increased in patients with NFAA after shifting to 6 months (HR, 1.41; 95% CI, 1.36-1.46; aHR, 1.19; 95% CI, 1.14-1.24; *P* < .001 in both) and 12 months (HR, 1.40; 95% CI, 1.35-1.46; aHR, 1.19; 95% CI, 1.14-1.24; *P* < .001 in both).

## Discussion

This large population-based study addressed NFAA and encompassed 17 726 individuals with NFAA and 124 366 controls. The results suggested increased overall mortality, cancer mortality, and cardiovascular mortality in those with an NFAA. Moreover, NFAA among younger individuals, as well as those not undergoing adrenalectomy, were associated with a higher relative mortality risk.

Previous studies in patients with AIs have found increased mortality in individuals with higher cortisol levels, mainly due to cardiovascular diseases.^5,9,14,15^ However, contrary to the present study, these studies did not exclude individuals with ACS. Moreover, only a few of the previous studies enrolled a control group without known adrenal masses. Taya et al^18^ performed a retrospective cohort study with 969 patients with AI and 2907 controls. This study demonstrated higher all-cause mortality among those with AI compared with those without (aHR, 1.14; 95% CI, 1.003-1.29). Exploratory analyses, limited by missing covariates, found that AIs were associated with an increased incidence of cancer (aHR, 1.61), diabetes, heart failure, peripheral vascular disease, kidney disease, and chronic pulmonary disease compared with controls. However, only 2.8% underwent at least 1 biochemical test on adrenal function, and only 0.4% underwent evaluation of all 3 adrenal axes, so it is not known if the adrenal lesions were nonfunctioning.

A recently published multicenter study by Deutschbein et al^19^ included 4374 patients with AI who were divided into 3 different groups according to cortisol levels after undergoing a dexamethasone suppression test (DST). During a follow-up of 7 years, 352 (9.6%) had died. All-cause mortality was significantly increased in patients with possible ACS (HR, 1.52; 95% CI, 1.19-1.94) and the ACS group (HR, 1.77; 95% CI, 1.20-2.62) compared with patients with nonfunctioning AI. In women younger than 65 years, a particularly high all-cause mortality was observed in those with ACS (HR, 4.39; 95% CI, 1.93-9.96).

To our knowledge, studies showing higher mortality risk due to cancer in patients with NFAA are lacking. Our group previously reported a higher mortality rate in ACS due to malignancy.^15^ However, the cohort was rather small since only 16 of 365 patients died of cancer (4.4%), 6 of them had cortisol levels after undergoing a DST that were consistent with ACS (ie, >138 nmol/L), and 5 of them had levels consistent with possible ACS while the rest had normal cortisol secretion in conjunction with cancer.

Di Dalmazi et al^5^ showed an increased mortality rate in patients with AI and higher cortisol levels. During follow-up, 9 of 21 individuals (43%) died due to cancer. The frequency of deaths attributable to cancer did not differ between the different groups of cortisol secretion. A recent study by Kjellbom et al^14^ showed similar results. In this study, 51 of 170 patients (30%) died due to malignancy. The cohort was divided into 4 groups according to cortisol levels after DST. Mortality due to cancer was similar in all the groups.

The current study showed an increased overall mortality, as well as mortality due to nonadrenal cancer and cardiovascular diseases, in patients with NFAA compared with controls. However, the underlying reason remains to be further elucidated. Several hypothetical explanations are possible. First, NFAA at incident may be hormonally inactive, but with time, they start secreting slightly elevated amount of cortisol, which can be associated with increased mortality. However, cortisol levels may already fluctuate at the incident, which makes NFAA not so nonfunctional. This hypothesis was already supported by previous authors.^1^ Increased mortality due to cardiovascular diseases in the current study potentially strengthens this hypothesis. Second, patients with NFAA could undergo more frequent radiological follow-up, which can be associated with greater rates of nonadrenal cancer. However, this still cannot explain increased mortality due to cancer. Moreover, the formation of tumors is generally facilitated by abnormal expression of growth-related genes.^20^ Thus, if an adrenal tumor is found, the same factors promoting its growth can be associated with the growth of other tumors^18^ (ie, the baseline tumorigenesis risk in the patients with NFAA was probably increased compared with controls). Finally, other undetected factors that coincide with NFAA may contribute to the increased risk of mortality.

To our knowledge, the current study was the first to show significantly higher overall mortality in patients with NFAA, as 23% of all deaths were associated with cancer. We speculated that some patients who underwent computed tomography imaging did so due to symptoms of cancer, which could contribute to a higher frequency of detected cancers. It could also be possible that NFAAs that were found on computed tomography images were metastases of not yet diagnosed cancer. To avoid confounding by indication, we excluded all cancer cases found within 3, 6, and 12 months in sensitivity analyses of the adrenal tumor diagnosis. Despite this, all-cause mortality remained higher compared with controls in all analyses. To support the study results, we conducted further sensitivity analyses for 2 groups of diseases for which imaging can be assumed to have been conducted for both cases and controls (ie, for cases and controls with acute appendicitis and cases and controls with gallbladder, biliary tract, and pancreas diseases). In acute appendicitis, imaging can also be assumed to have been performed without suspicion of another illness or cancer. Moreover, acute appendicitis was not expected to be associated with increased mortality, whereas gallbladder, biliary tract, and pancreas diseases may have. In both analyses, we expected similar baseline mortality in cases and controls, so any differences in mortality could be attributable to NFAA. Overall, in both analyses, mortality was still significantly increased in cases (ie, in individuals with NFAA).

The results of this study also suggested that the association between NFAA and mortality was most pronounced in individuals younger than 65 years. This finding was unexpected and so far has no clear explanation. Unlike most hormones, whose levels diminish throughout aging, mean cortisol secretion tends to increase.^21,22^ These changes in cortisol secretion are associated with impaired cognitive status, dementia, anxiety, and depression that is associated with aging.^21^ The previously published studies promote the idea that gradually rising cortisol levels with age are somehow physiological. One could assume that presence of NFAA at a young age is associated with slightly higher cortisol secretion, which could be associated with earlier development of these changes, which in turn may be associated higher mortality that would relatively affect younger people more since their overall mortality risk is lower (ie, they have fewer competing causes of mortality). Moreover, some of these NFAAs may later start producing mild excess of cortisol, and this risk may be higher in younger individuals since they have more time to develop cortisol excess.

In the current study, those few patients (n = 352) who underwent adrenalectomy had similar mortality as controls, while those who did not undergo an adrenalectomy had higher mortality than controls. It may be that healthier patients tend to be selected to undergo adrenalectomy, which is associated with better survival. However, it could be speculated that after adrenalectomy, these mildly unphysiological cortisol levels become physiological, which can be associated with a longer survival. Randomized clinical trials are needed to confirm this hypothesis.

In 2016, the European Society of Endocrinology published guidelines on the management of AI.^3^ The guidelines not only suggested against repeated hormonal workup in patients with AI with normal hormonal secretion at initial evaluation, but also in those with a possible ACS, if comorbidities potentially associated with hypercortisolism, such as hypertension and type 2 diabetes, were absent. Keeping in mind further findings, follow-up was questioned, and prolonged follow-up time was suggested.^23^ The latest guidelines of the American Association of Endocrine Surgeons suggest hormonal reevaluation at a 2-year to 5-year interval.^24^ However, it is unclear if the patients with NFAA would benefit from greater follow-up.

### Limitations

This study had several limitations. We did not have access to the hormonal evaluation of the patients or radiological reports. Consequently, some individuals may have received an NFAA diagnosis despite having slightly elevated cortisol levels. Moreover, it can be assumed that patients who have cancer-related symptoms undergo radiological imaging more frequently, and even if we tried to account for that by excluding all those with a cancer diagnosis within first 3 months after the NFAA diagnosis (and 6 and 12 months in the sensitivity analyses), this may still not have been enough. Furthermore, although we adjusted for several potential confounders, such as comorbidities and socioeconomic factors, residual confounding cannot be excluded. For example, the difference between cases and controls for some comorbidities (eg, chronic obstructive pulmonary disease) was quite large, and despite adjustment, this may have affected results. However, the sensitivity analyses confirmed the main results.

## Conclusion

In this case-control study, NFAA was associated with an increased overall mortality and mortality due to cardiovascular disease and cancer. It was more pronounced among younger individuals. Those who underwent adrenalectomy had no increased mortality.

## Notes

Supplement 1. eFigure 1. Overall survival by sex eFigure 2. Overall survival by age eFigure 3. Overall survival by adrenalectomy eFigure 4. Overall survival in sensitivity analyses eTable. Potential confounders

Supplement 2. Data sharing statement
