# Supplementary material for: Mortality in Patients With Nonfunctional Adrenal Tumors
Source: JAMA Intern Med. 2023 Jun 26;183(8):832–8. doi: 10.1001/jamainternmed.2023.2442 (PMC10294015; doi:10.1001/jamainternmed.2023.2442)
Supplement: Supplement 1. — eFigure 1. Overall survival by sex eFigure 2. Overall survival by age eFigure 3. Overall survival by adrenalectomy eFigure 4. Overall survival in sensitivity analyses eTable. Potential confounders [file jamainternmed-e232442-s001.pdf]

Supplemental Online Content

Patrova J, Mannheimer B, Lindh JD, Falhammar H. Mortality in patients with nonfunctional adrenal tumors. *JAMA Intern Med*. Published online June 26, 2023. doi:10.1001/jamainternmed.2023.2442

eFigure 1. Overall survival by sex

eFigure 2. Overall survival by age

eFigure 3. Overall survival by adrenalectomy

eFigure 4. Overall survival in sensitivity analyses

eTable. Potential confounders

This supplemental material has been provided by the authors to give readers additional information about their work.

© 2023 Patrova J et al. *JAMA Internal Medicine.*

eFigure 1. Overall survival by sex: Panel A- women, Panel B- men. Kaplan-Meier survival curves after post-hoc analysis showing mortality in subgroups of the 17,726 individuals with non-functional adrenal adenomas compared to 124,366 controls, Cox-proportional hazard ratio analysis, adjusted for age and possible confounders, P <0.001. All individuals with malignancy discovered within the first three months after the diagnosis of the non-functional adrenal adenoma have been excluded.

A:

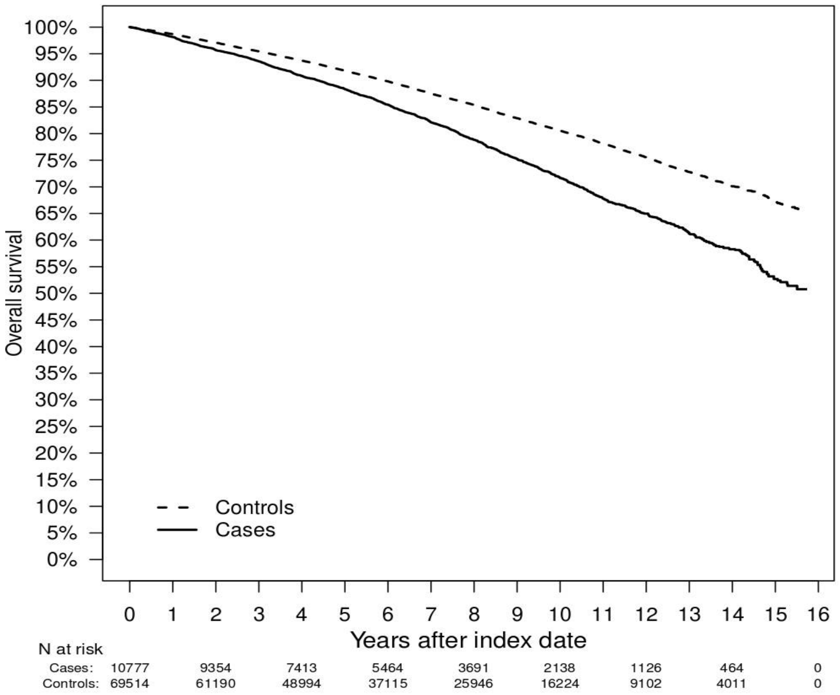

B:

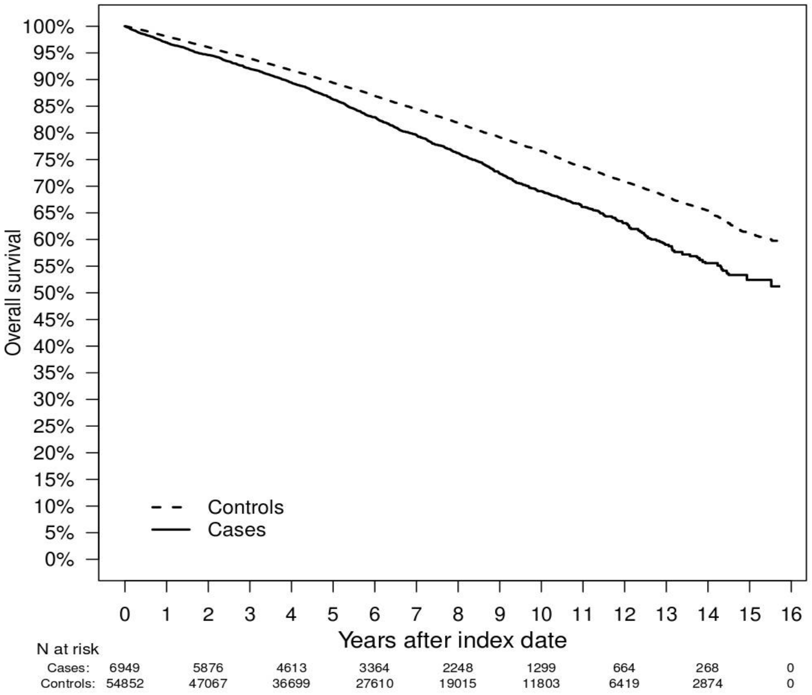

© 2023 Patrova J et al. *JAMA Internal Medicine.*

eFigure 2. Overall survival by age: Panel A: < 65-year-old, Panel B: ≥ 65-year-old. Kaplan-Meier survival curves after post-hoc analysis showing mortality in subgroups of the 17,726 individuals with non-functional adrenal adenomas compared to 124,366 controls, Cox-proportional hazard ratio analysis, adjusted for age and possible confounders, P <0.001. All individuals with malignancy discovered within the first three months after the diagnosis of the non-functional adrenal adenoma have been excluded.

A:

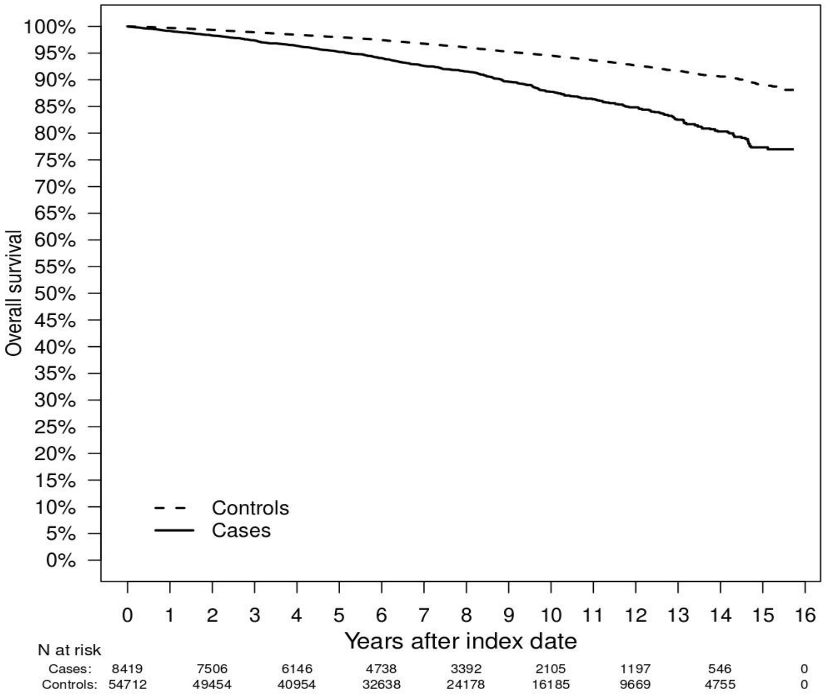

B:

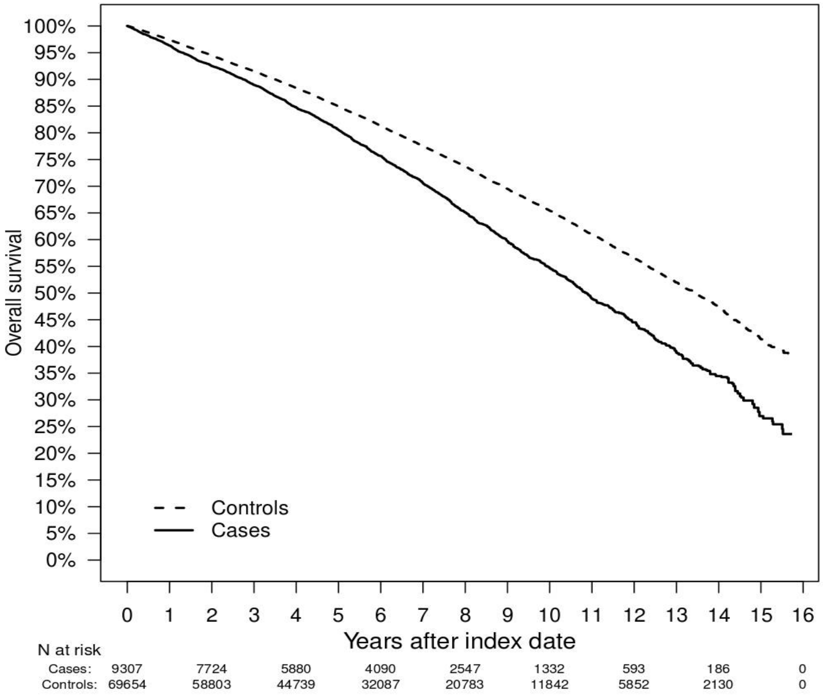

© 2023 Patrova J et al. *JAMA Internal Medicine.*

eFigure 3. Overall survival by adrenalectomy: Panel A: no adrenalectomy, Panel B: adrenalectomy. Kaplan-Meier survival curves after post-hoc analysis showing mortality in subgroups of the 17,726 individuals with non-functional adrenal adenomas compared to 124,366 controls, Cox-proportional hazard ratio analysis, adjusted for age and possible confounders, P-value in Panel A <0.001, P-value in Panel B = 0.03. All individuals with malignancy discovered within the first three months after the diagnosis of the non-functional adrenal adenoma have been excluded.

A:

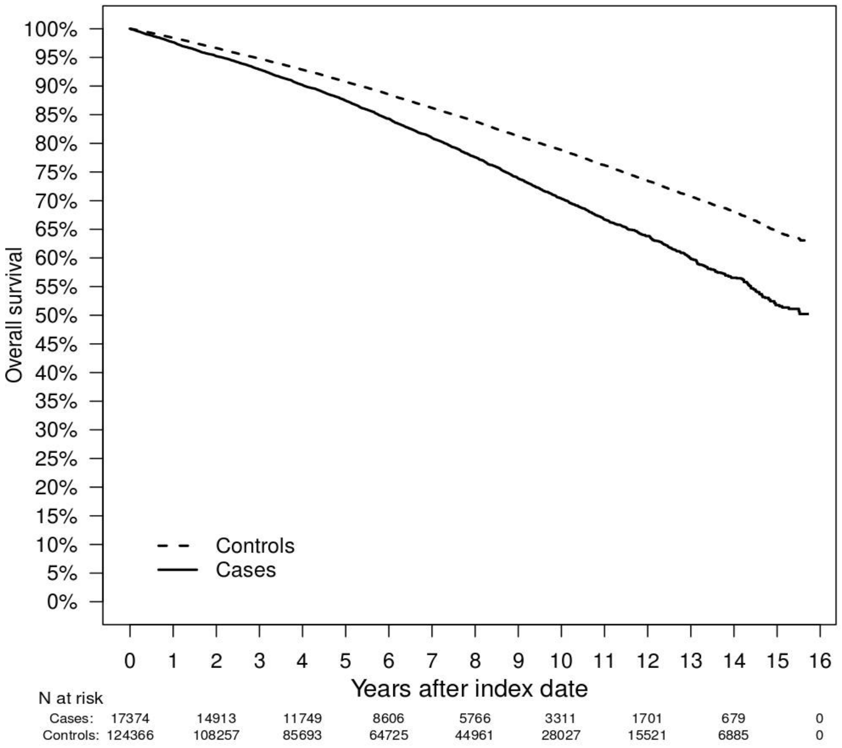

B:

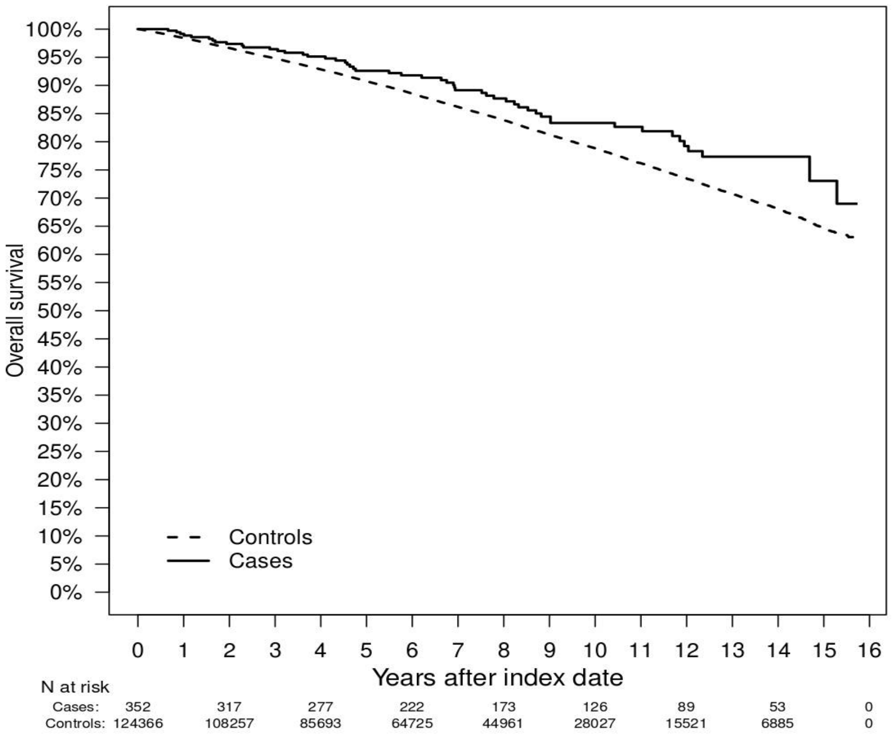

© 2023 Patrova J et al. *JAMA Internal Medicine.*

eFigure 4. Overall survival in sensitivity analyses: Panel A: acute appendicitis, Panel B: gallbladder, biliary tract and pancreas diseases. Panel A: Kaplan-Meier survival curves after sensitivity analysis showing mortality in 155 individuals with appendicitis and non-functional adrenal adenomas compared to 2,154 controls with appendicitis. Panel B: Kaplan-Meier survival curves after sensitivity analysis showing mortality in 802 individuals with non-functional adrenal adenomas and gallbladder and/or biliary tract and/or pancreas diseases compared to 10,213 controls with gallbladder and/or biliary tract and/or pancreas disease. P-value in Panel A and B <0.001. All individuals with malignancy discovered within the first three months after the diagnosis of the non-functional adrenal adenoma have been excluded.

A:

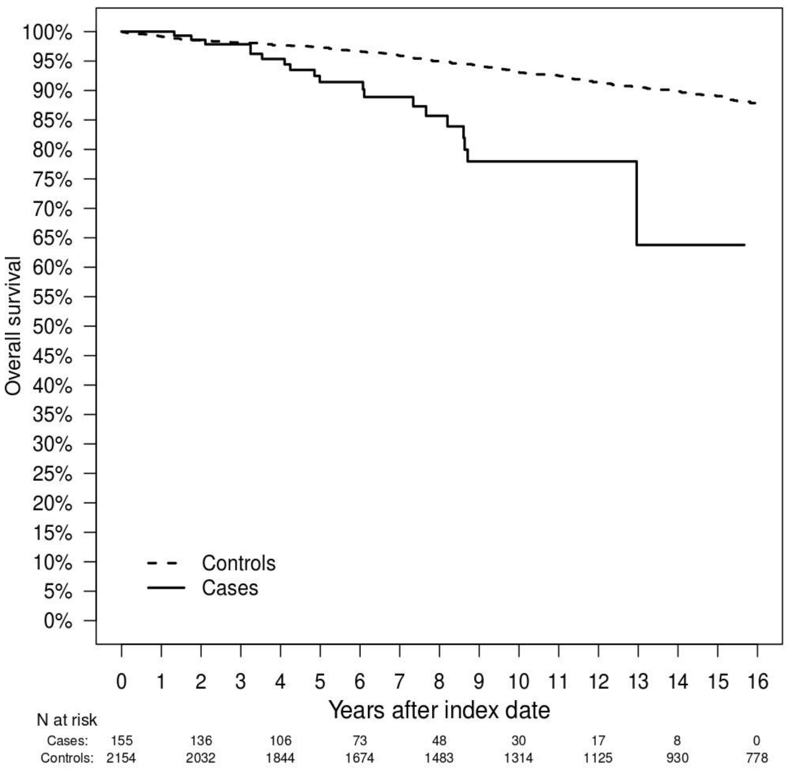

B:

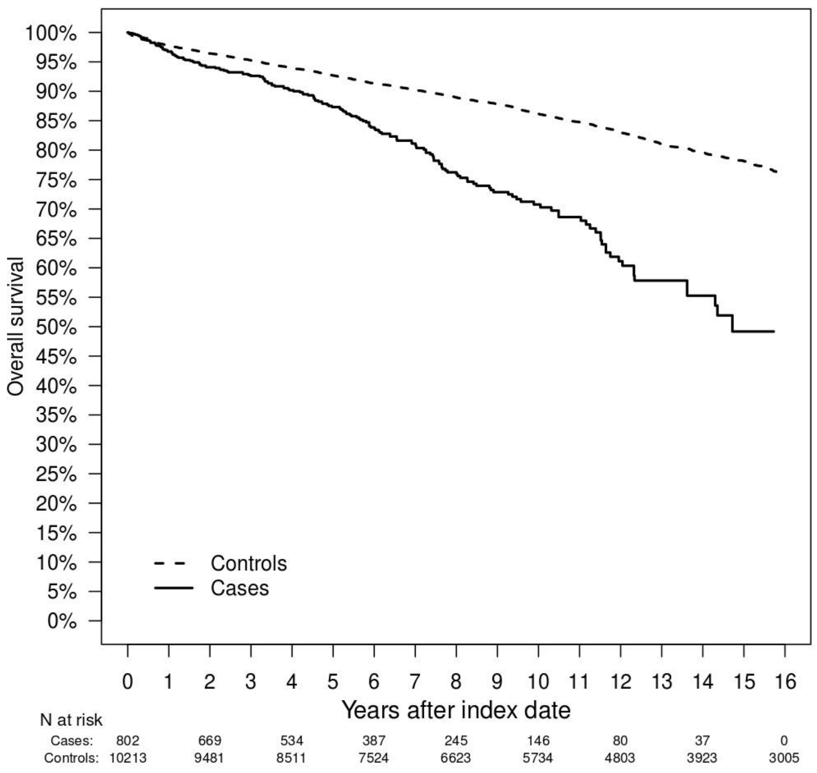

© 2023 Patrova J et al. *JAMA Internal Medicine.*

eTable 1. Potential confounders

Confounder	Composite variables	ICD-10 code
**Renal diseases**	Renal failure	N17-19
**Infections**	Sepsis<br>Pneumonia<br>Meningitis, encephalitis, brain abscess	A41<br>J18<br>G00 - G07
**Cardiovascular diseases**	Ischemic heart disease<br>Heart failure<br>Thromboembolism	I20-I25<br>I50<br>I802 and I26
**Gastrointestinal diseases**	Gallbladder, biliary tract and pancreas diseases<br>Inflammatory bowel disease<br>Liver failure / liver diseases	K80-K87<br><br>K51, K50<br>K70-77
**Cerebrovascular diseases**	Nontraumatic subarachnoid hemorrhage<br>Nontraumatic intracerebral hemorrhage<br>Other and unspecified nontraumatic intracranial hemorrhage<br>Cerebral infarction<br>Stroke, not specified as hemorrhage or infarction<br>Sequelae of cerebrovascular disease	I60<br>I61<br>I62<br><br>I63<br>I64<br>I69
**Other diseases**	Chronic obstructive pulmonary disease / Pulmonary emphysema	J44 and/or J43
**Alcohol misuse**		E24.4, F10, G62.1, G72.1, I42.6, K29.2, K70, K86
**Socioeconomic factors**	Education:<br>Preschool education<br>Preschool class education<br>Primary or lower secondary education<br>Upper secondary education<br>Bachelor’s level education<br>Master’s level education<br>Postgraduate education<br><br>Income (Disposable income for one person in SEK)<br><br>Unemployment (Number of days in unemployment)	
**Number of prolonged hospitalizations**	≥3 days hospitalization at least once	

© 2023 Patrova J et al. *JAMA Internal Medicine.*
