# Supplementary material for: Mortality in Patients With Nonfunctional Adrenal Tumors
Source: JAMA Intern Med. 2023 Jun 26;183(8):832–8. doi: 10.1001/jamainternmed.2023.2442 (PMC10294015; doi:10.1001/jamainternmed.2023.2442)
Supplement: Supplement 2. — Data sharing statement [file jamainternmed-e232442-s002.pdf]

Data Sharing Statement

Patrova. Mortality in Patients with Nonfunctional Adrenal Tumors. *JAMA Intern Med*. Published June 26, 2023. doi:10.1001/jamainternmed.2023.2442

Data
Data available: Yes
Data types: Data (not involving human participants)
How to access data: jekaterina.patrova@ki.se
When available: With publication

Supporting Documents
Document types: None

Additional Information
Who can access the data: Researchers whose proposed use of the data has been approved.
Types of analyses: For a specified purpose
Mechanisms of data availability: After approval of a proposal, with a signed data access agreement
